# Ca^2+^ Extrusion by NCX Is Compromised in Olfactory Sensory Neurons of OMP^−/−^ Mice

**DOI:** 10.1371/journal.pone.0004260

**Published:** 2009-01-23

**Authors:** Hyun J. Kwon, Jae Hyung Koo, Frank Zufall, Trese Leinders-Zufall, Frank L. Margolis

**Affiliations:** 1 Department of Anatomy and Neurobiology, School of Medicine, University of Maryland, Baltimore, Maryland, United States of America; 2 Department of Engineering and Computer Science, Andrews University, Berrien Springs, Michigan, United States of America; 3 Department of Physiology, University of Saarland, Homburg, Germany; Emory University, United States of America

## Abstract

**Background:**

The role of olfactory marker protein (OMP), a hallmark of mature olfactory sensory neurons (OSNs), has been poorly understood since its discovery. The electrophysiological and behavioral phenotypes of OMP knockout mice indicated that OMP influences olfactory signal transduction. However, the mechanism by which this occurs remained unknown.

**Principal Findings:**

We used intact olfactory epithelium obtained from WT and OMP^−/−^ mice to monitor the Ca^2+^ dynamics induced by the activation of cyclic nucleotide-gated channels, voltage-operated Ca^2+^ channels, or Ca^2+^ stores in single dendritic knobs of OSNs. Our data suggested that OMP could act to modulate the Ca^2+^-homeostasis in these neurons by influencing the activity of the plasma membrane Na^+^/Ca^2+^-exchanger (NCX). Immunohistochemistry verifies colocalization of NCX1 and OMP in the cilia and knobs of OSNs. To test the role of NCX activity, we compared the kinetics of Ca^2+^ elevation by stimulating the reverse mode of NCX in both WT and OMP^−/−^ mice. The resulting Ca^2+^ responses indicate that OMP facilitates NCX activity and allows rapid Ca^2+^ extrusion from OSN knobs. To address the mechanism by which OMP influences NCX activity in OSNs we studied protein-peptide interactions in real-time using surface plasmon resonance technology. We demonstrate the direct interaction of the XIP regulatory-peptide of NCX with calmodulin (CaM).

**Conclusions:**

Since CaM also binds to the Bex protein, an interacting protein partner of OMP, these observations strongly suggest that OMP can influence CaM efficacy and thus alters NCX activity by a series of protein-protein interactions.

## Introduction

Olfactory marker protein (OMP) is an abundant cytosolic protein whose expression is highly restricted to mature OSNs in all vertebrates [1]–[8]. Although of unknown function, it has been implicated to play a role in olfactory transduction [9]–[15]. Homozygous OMP knockout (OMP^−/−^) mice exhibit strongly reduced onset and decay kinetics of odor responses, resulting in delayed recovery following odor stimulation [10], [15]. Changes in the response kinetics of olfactory sensory neurons (OSNs) have previously been shown to depend on changes in intracellular Ca^2+^ concentration [16]–[20], leading to the hypothesis that OMP could be involved in modulating the Ca^2+^ homeostasis in these neurons.

The initial steps leading to Ca^2+^ entry in vertebrate OSNs begin in the cilia when odor molecules bind to specific receptors and initiate a cascade of events leading to rapid cAMP formation, followed by the opening of cyclic nucleotide-gated (CNG) cation channels [21]–[26]. The considerable Ca^2+^ permeability of CNG channels [27], [28] causes a rapid intraciliary Ca^2+^ increase [19], [29], [30] which, in turn, causes further excitation through subsequent activation of Ca^2+^-dependent Cl^−^ channels[31]–[35]. The Ca^2+^ rise also mediates adaptation by modulating CNG channel activity [17], [36] and Ca^2+^/calmodulin kinase II-dependent attenuation of adenylyl cyclase activity [18], [20].

Despite a recent report suggesting that OMP plays a modulatory role in cAMP formation [9], OMP might play additional roles to influence a common regulator of several steps in the transduction cascade. One possible target could be calmodulin (CaM) that is Ca^2+^-dependent and a critical effector at several steps of olfactory transduction. Interestingly, Bex protein, an interacting partner of OMP [37]–[40], binds CaM in a Ca^2+^-dependent manner [41], providing the basis for a potential mechanistic link among OMP, Bex and CaM with various players of the olfactory signal transduction cascade.

To investigate the possibility that OMP participates in modulating Ca^2+^ homeostasis, we imaged Ca^2+^ dynamics of single olfactory knobs in wild-type and OMP^−/−^ mice. Ca^2+^responses were induced by activating and blocking various pathways involved in Ca^2+^ influx, sequestration, and extrusion. We present here evidence that OMP facilitates NCX activity and allows rapid Ca^2+^ extrusion from OSN knobs. Furthermore, our protein interaction data suggest that the influence of OMP on NCX activity may be mediated through its interactions with Bex protein and calmodulin.

## Results

**Figure 1:**
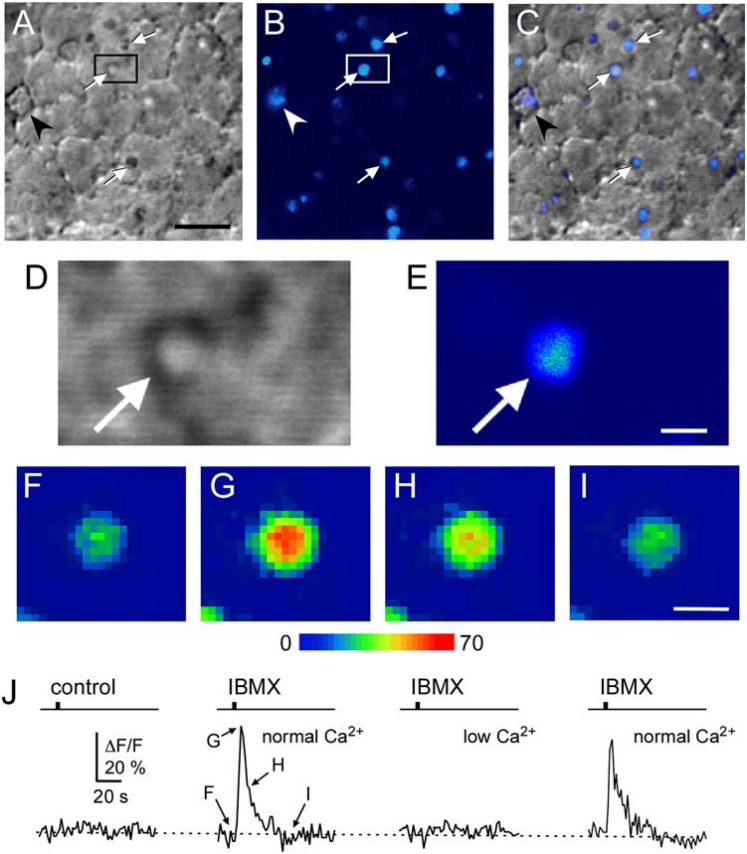
Imaging Ca^2+^-transients in single dendritic knobs of mouse OSNs *in situ*. A–B, Low resolution transmitted light (A) and confocal fluoresence image in pseudocolor (B) of the olfactory epithelium preparation at rest. Scale bar, 5 µm. Multiple olfactory knobs are clearly visible (white arrows). A single Bowman gland (arrowhead) is also identifiable based on its size and shape. C, Resting fluorescence Ca^2+^ signal superimposed onto the anatomical map. D, E, High resolution transmitted light (D) and confocal fluorescence image (E) of the olfactory knob delimited by the black box in A (arrow). F–I, Time series images of the same knob shown at rest (F), after focal application of a 1-s IBMX pulse (100 µM) (G–H), and following recovery of the fluorescence signal (I). The time points at which these images were acquired are indicated in (J). J, Analysis of the time course of the IBMX-evoked Ca^2+^ signal. The response was reversibly abolished by lowering the external Ca^2+^ concentration from 1 mM (normal Ca^2+^) to 0.6 µM (low Ca^2+^).

To test whether OMP is involved in Ca^2+^ signaling and to determine where in the signal transduction cascade OMP might be acting, we compared the response kinetics of the Ca^2+^ transients elicited in OSN knobs of WT and OMP^−/−^ mice by confocal Ca^2+^ imaging. All elements required during olfactory signal transduction have been reported to be present in both the cilia and knob [42], [43], except for the olfactory receptors that are only present on OSN knobs during development [44], [45]. The primary source of Ca^2+^ entry during the olfactory signal transduction cascade are the cyclic-nucleotide gated (CNG) channels in OSN cilia [29]. These CNG channels are not only highly enriched in the ciliary compartment, but also at the olfactory knob [46], [47] and have been shown to be to a high degree the source of Ca^2+^ increases seen in this OSN compartment too [19], [29], [48]. Visualization of Ca^2+^ dynamics in the mouse olfactory knob could be achieved at excellent spatial and temporal resolution using confocal imaging (Figure 1), but unlike previous experiments using salamander OSNs [19], [29], [48] we were unable to visualize stimulus-induced Ca^2+^ signals in individual cilia under these conditions. The knobs were identified by their characteristic circular shape and size (∼1 µm) observed in both the transmission (Figure 1A, D) and the confocal fluorescence image (Figure 1B, C, E). The dynamics of the Ca^2+^ signal occurring in the knob of WT OSNs in response to receptor-independent elevation of the intracellular cAMP concentration, induced by a brief focal pulse of the phosphodiesterase inhibitor IBMX (100 µM), were captured (Figure 1F–I) and time courses analyzed (Figure 1J). The IBMX-induced Ca^2+^ transient was abolished reversibly by lowering the external Ca^2+^ concentration in the bath solution from 1 mM (normal Ca^2+^) to 0.6 µM (low Ca^2+^) (Figure 1J), demonstrating that this Ca^2+^ rise was caused primarily by Ca^2+^ entry. This experiment also shows that multiple evoked Ca^2+^ transients could be recorded from a single knob upon repetitive stimulation under various ionic manipulations. Unless otherwise stated, subsequent experiments were performed using the same basic protocol.

### Slower recovery kinetics of Ca^2+^ transients in OSNs of OMP^−/−^ mice

**Figure 2:**
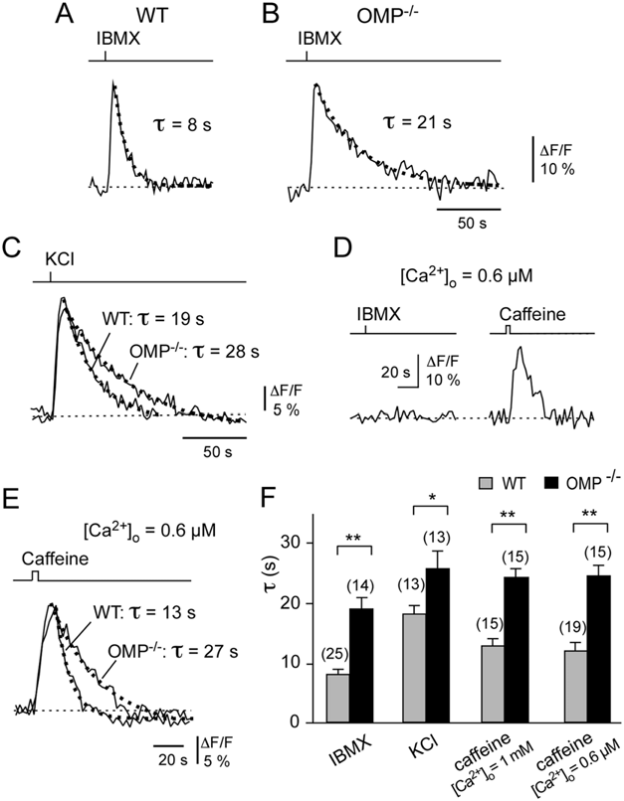
Recovery of elevated intracellular Ca^2+^ levels is compromised in OMP^−/−^ mice. A–C, Comparison of fluorescence intensity changes (ΔF/F) of Ca^2+^ responses in WT and OMP^−/−^ knobs to a 1-s pulse of 100 µM IBMX (A, B) or 80 mM KCl (C) using 1 mM external Ca^2+^. Recovery time course of the signals was fitted with single exponential functions (dashed lines). The decay time constants (τ) of the fitted curves are indicated. These results demonstrate an apparent defect in the kinetics of removal of elevated Ca^2+^_i_ from the dendritic knobs of the OSNs of OMP^−/−^ mice. D, Ca^2+^ response of a single WT knob stimulated with a 1-s pulse of IBMX (100 µM) followed by a 5-s pulse of caffeine (10 mM), both in low external Ca^2+^ solution (0.6 µM), confirming that the caffeine-induced Ca^2+^ transient depends on an intracellular source. E, Comparison of Ca^2+^ transients in WT and OMP^−/−^ knobs evoked by a 5-s pulse of caffeine (10 mM) in low extracellular Ca^2+^ solution (0.6 µM). Recovery time course of the signals was fitted with single exponential functions (dashed lines). The decay time constants (τ) of the fitted curves are indicated. F, Bar graphs showing collected results from OSN knobs of WT and OMP^−/−^ mice. For stimulation with IBMX, WT: τ = 8.5±1.3 s (*n* = 25, *N* = 8), OMP^−/−^: τ = 21.3±2.2 s (*n* = 13, *N* = 4). For stimulation with KCl, WT: τ = 20.1±1.7 s (*n* = 13, *N* = 4), OMP^−/−^: τ = 28.7±3.2 s (*n* = 13, *N* = 4). For stimulation with caffeine in 1 mM Ca^2+^_o_, WT: τ = 13.9±1.6 s (*n* = 15, *N* = 5), OMP^−/−^: τ = 26.8±1.9 s (*n* = 15, *N* = 4), **p<0.0001 and in low external Ca^2+^ (0.6 µM), WT: τ = 13.1±1.8 s (*n* = 19, *N* = 6), OMP^−/−^: τ = 27.3±1.9 s (*n* = 15, *N* = 5), *p<0.001; **p<0.0001.

To determine whether Ca^2+^ signaling differs in knobs of OMP^−/−^ *vs*. WT OSNs, we first compared the Ca^2+^ transients evoked by a 1-s pulse of IBMX (100 µM) (Figure 2A, B). Analysis of the kinetics of the resulting Ca^2+^ transients indicated that there was no significant difference in the onset of the signal (the time-to-peak: WT: 5.9±0.7 s, number of knobs, *n* = 25, number of animals, *N* = 8; OMP^−/−^: 6.7±1.4 s, *n* = 13, *N* = 4). By contrast, the recovery kinetics of the Ca^2+^ signal back to baseline were significantly altered between WT and OMP^−/−^ (*p*<0.0001). Fits of the single-exponential decay yielded a time constant (τ) that gave a measure of the Ca^2+^ recovery rate. On average, the decay time constant of an IBMX-evoked Ca^2+^ transient was approximately 2.5-fold slower in OMP^−/−^ *vs*. WT knobs (Figure 2A, B, F). Hence, OMP^−/−^ OSNs show a pronounced deficit in eliminating Ca^2+^ that has entered the cell following cAMP formation and CNG channel gating. This finding supports our general hypothesis that OMP participates in modulation of Ca^2+^ signaling in OSN knobs. IBMX application bypasses the initial steps of the olfactory signal transduction cascade (ligand-receptor binding, and G-protein activation of adenylyl cyclase), therefore, the deficit in Ca^2+^ recovery kinetics is attributed to a compromised ability of events subsequent to cAMP generation, e.g. inefficient adaptation of CNG channels allowing Ca^2+^ entry for a longer period of time or a defect in the Ca^2+^ clearance processes.

Since Ca^2+^ influx through CNG channels is an important factor in determining the overall Ca^2+^ flux, we next asked whether there could be altered feedback inhibition of CNG channels due to elevated resting Ca^2+^_i_ in the OMP^−/−^ mouse. Resting endogenous Ca^2+^_i_ levels in OSNs were measured using calibration procedures with either fluo-4 or fura-2. From single wavelength calibration with fluo-4, the free Ca^2+^_i_ at rest was estimated to be 76.3±3.3 nM (*n* = 16, *N* = 6) in WT knobs and 79.4±4.3 nM (*n* = 10, *N* = 4) in OMP^−/−^ knobs. Based on ratiometric calibration with fura-2, resting levels of Ca^2+^_i_ were calculated to be 83.0±13.7 nM in WT (*n* = 13, *N* = 4) and 87.1±15.6 nM (*n* = 9, *N* = 3) in OMP^−/−^ knobs. In neither measurement was there any significant difference in Ca^2+^_i_ resting levels between WT and OMP^−/−^ mice, arguing against the possibility of altered inhibition of CNG channels due to elevated Ca^2+^_i_ in OMP^−/−^ mice. To determine whether the reduced Ca^2+^ recovery rate in OMP^−/−^ mice was dependent on the source of Ca^2+^-entry, we tested the effects of raising Ca^2+^_i_ by membrane depolarisation which leads to the opening of voltage-operated Ca^2+^ channels, or by releasing Ca^2+^ from intracellular stores [48], [49]. Therefore, Ca^2+^ transients evoked by application of KCl or caffeine were monitored in subsequent experiments. We found that Ca^2+^ transients elicited by a 1-s pulse of KCl (80 mM) showed an approximately 1.5-fold slower recovery time constant in OMP^−/−^ knobs compared to WT controls (*p*<0.001) (Figure 2C, F). Thus, a reduced Ca^2+^ recovery rate in OMP^−/−^ knobs was observed irrespective of whether the Ca^2+^ transient was elicited by the opening of CNG- or voltage-gated Ca^2+^ channels, consistent with the hypothesis that OMP^−/−^ OSN knobs exhibit a deficit in Ca^2+^ removal mechanisms. This was further supported in experiments using caffeine, a ryanodine receptor agonist that depletes Ca^2+^ from a releasable pool of intracellular Ca^2+^ stores [48]. Pulsed application of caffeine (5 s, 10 mM) evoked a Ca^2+^ transient in olfactory knobs of both genotypes, but the recovery rate was significantly slower in the OMP^−/−^ knobs, by approximately 2-fold (*p*<0.0001; Figure 2E, F). Importantly, these latter experiments were performed under reduced external Ca^2+^ (0.6 µM), a condition under which IBMX failed to induce a measurable Ca^2+^ rise (Figure 2D). This shows that the caffeine-evoked Ca^2+^ signal was derived solely from intracellular sources, demonstrating that mammalian olfactory knobs contain substantial amounts of releasable intracellular Ca^2+^ stores. The same phenotype was also observed at normal (1 mM) external Ca^2+^ (Figure 2F). Together, these results demonstrate that OMP^−/−^ OSN knobs are impaired in Ca^2+^ recovery kinetics even when elevated Ca^2+^_i_ resulted from emptying intracellular stores. Thus, under conditions where Ca^2+^_i_ was elevated by activating any one of three different mechanisms, slower Ca^2+^ recovery kinetics were reproducibly observed, indicating that the phenomenon was independent of the source of Ca^2+^. Together, these results demonstrate an apparent defect in the kinetics of removal of elevated Ca^2+^_i_ from the dendritic knobs of the OSNs of OMP^−/−^ mice.

### NCX provides a major Ca^2+^ extrusion mechanism in OSN knobs

The observed delay in restitution kinetics of the Ca^2+^_i_ transients of OMP^−/−^ mice indicates that there is a defect in the Ca^2+^ clearance mechanism in the OSN knobs. In mammalian cells, elevated Ca^2+^_i_ can be reduced by sequestering it into Ca^2+^ stores through sarco-endoplasmic-reticulum Ca^2+^-ATPase (SERCA) pumps or by expelling Ca^2+^ through the plasma membrane by a Na^+^/Ca^2+^ exchanger (NCX) and/or plasma membrane Ca^2+^-ATPases (PMCA). Using electrophysiological criteria it has been reported that NCX is the major means of Ca^2+^ extrusion following odor-evoked stimulation of the apical region of amphibian and mouse OSNs [50], [51]. The demonstration of NCX1 in isolated cilia by immunoblotting [52] is consistent with these reports. This has not been previously analyzed using Ca^2+^ imaging of mouse OSN knobs. Recent reports suggests that PMCA may also play a significant role in Ca^2+^ removal in the rat [52] and mouse [53] OSNs. Therefore, we evaluated the contribution of each Ca^2+^ clearance mechanism to the removal of elevated Ca^2+^_i_.

**Figure 3:**
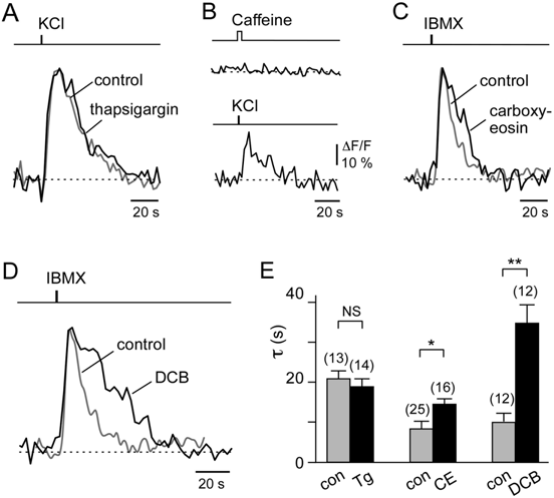
NCX is critical for reducing elevated intracellular Ca^2+^ levels in OSN knobs. A, Comparison of the kinetic properties of the Ca^2+^ transients (normalized responses) in OSN knobs evoked by a 1-s pulse of KCl (80 mM) before (control) and after thapsigargin (200 nM) treatment. B, Ca^2+^ response (ΔF/F) of a single knob stimulated with a 5-s pulse of caffeine (10 mM) followed by a 1-s pulse of KCl (80 mM), confirming that thapsigargin (200 nM) pretreatment depleted Ca^2+^ from intracellular stores. C, Comparison of the kinetic properties of the Ca^2+^ transients (normalized responses) in OSN knobs evoked by a 1-s pulse of IBMX (100 µM) before (control) and after treatment with the PMCA inhibitor carboxyeosin (10 µM). D, Comparison of the kinetic properties of IBMX-induced Ca^2+^ transients (normalized responses) in OSN knobs before (control) and after treatment with the NCX inhibitor 3,4-dichlorobenzamil hydrochloride (DCB; 10 µM). E, Bar graphs showing collected decay time constants from control (gray) and treated (black) OSN knobs. For thapsigargin (Tg), control: τ = 21.1±1.9 s (*n* = 13, *N* = 4), Tg: τ = 19.0±1.9 s (*n* = 14, *N* = 4). For carboxyeosin (CE), control: τ = 8.5±1.9 s (*n* = 25, *N* = 8), CE: τ = 13.3±1.4 s (*n* = 16, *N* = 4). For 3,4-dichlorobenzamil hydrochloride (DCB), control: τ = 11.0±1.4 s (*n* = 12, *N* = 4), DCB: τ = 35.1±4.4 s (*n* = 12, *N* = 4). NS, not significant; *p<0.05; **p<0.0001. The Ca^2+^ transients in A,C, and D were rescaled to give the same peak amplitude.

Since mouse OSN knobs contain accessible Ca^2+^ stores, we next evaluated whether the SERCA pumps could play a significant role in Ca^2+^ removal in the WT OSNs. To test this, the decay rate constants of the depolarization-induced Ca^2+^ transients were characterized both before and after inhibition of the SERCA pumps with 200 nM thapsigargin (Tg) (Figure 3A). After treatment with Tg, the decay rate of the depolarization-induced Ca^2+^ transients remained unchanged in the WT knobs when the contribution of the SERCA pump was eliminated. We were not able to observe a caffeine-induced Ca^2+^ response (10 mM; *n* = 10; *N* = 3) following treatment with Tg (Figure 3B), confirming that the Tg treatment was sufficient to discharge the Tg-sensitive Ca^2+^ pool and impair the SERCA pumps irreversibly. These results demonstrate that the contribution of the SERCA pumps to eliminate the elevated Ca^2+^_i_ is negligible. When we tested OMP^−/−^ knobs in the Tg paradigm the Ca^2+^ recovery kinetics were unchanged subsequent to the Tg treatment (data not shown, *n* = 13; *N* = 4) confirming that SERCA pumps do not affect the recovery kinetics of Ca^2+^ responses of the OMP^−/−^ mice. These results suggest that the elimination of Ca^2+^_i_ from OSN knobs should be mediated mostly by plasma membrane-dependent Ca^2+^ extrusion mechanisms.

Therefore, we next determined the relative contributions of the plasma membrane Ca^2+^-ATPase and the Na^+^/Ca^2+^ exchanger to Ca^2+^_i_ removal. To achieve this, decay kinetics of Ca^2+^ transients were evaluated under conditions where each was selectively inhibited. In the presence of carboxyeosin (CE, 10 µM), a potent PMCA inhibitor [54], [55], the IBMX-induced Ca^2+^ transients showed, on average, less than 1.5-fold reduction in the recovery rate to basal level (Figure 3C, E). This result indicates that although PMCA does participate in Ca^2+^ removal from OSN knobs [52], [53] another mechanism is still required to account for removal of the bulk of the elevated Ca^2+^_i_.

To evaluate the contribution of plasma membrane NCX in reducing elevated Ca^2+^_i_ we tested the effect of the NCX inhibitor 3,4-dichlorobenzamil hydrochloride (DCB, 10 µM) on WT knobs. In the presence of DCB, the recovery rate of an IBMX-induced Ca^2+^ transient was considerably altered, increasing by approximately 3.8-fold (Figure 3D, E). When DCB was washed out, the kinetics of Ca^2+^_i_ recovery were restored close to those observed before inhibition (data not shown), demonstrating both the efficacy and reversibility of the DCB effect. Conversely, DCB has been reported to block CNG channels [56]–[58] and could affect the Ca^2+^ influx. We observed a slight decrease in maximal IBMX-induced fluorescence intensity during DCB treatment (control: F = 16.7±1.4%, n = 12; DCB: F = 13.8±1.5%, n = 12), however this decrease was not significant (t-test: p = 0.16) excluding a potential involvement of CNG channels. These results suggest, therefore, that OSN knobs use NCX as their primary Ca^2+^_i_ extrusion mechanism in response to stimulus-induced Ca^2+^_i_ elevation (Figure 3E). Furthermore it is consistent with reports from other laboratories [51], [59], [60] suggesting that NCX serves as a major Ca^2+^ extrusion pathway for Ca^2+^_i_ transients raised by odorant stimulation. Hence, the slower restitution kinetics in the OMP^−/−^ mice might be indicative of a deficit in NCX activity.

### NCX activity is compromised in OMP^−/−^ mice

**Figure 4:**
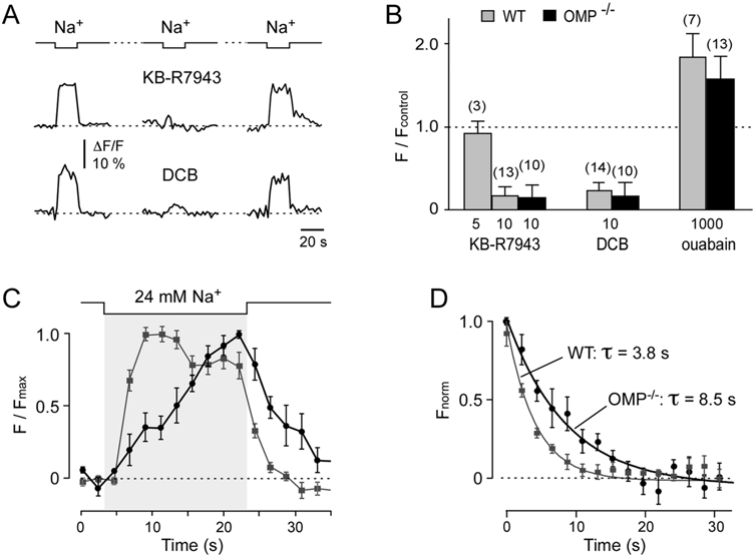
OMP facilitates NCX activity. A, The activity of NCX was probed in reverse mode by monitoring the rise of Ca^2+^_i_ in response to a stepwise reduction of Na^+^_o_ (by substituting Na^+^ by Li^+^). Ca^2+^ responses are reversibly inhibited by the NCX inhibitor KB-R7943 (10 µM) as well as by dichlorobenzamil (DCB, 10 µM). This confirms that the rise in Ca^2+^ induced by low Na^+^_o_ is a result of Ca^2+^ entry through NCX. B, Bar histogram of the Ca^2+^ responses obtained after treatment with pharmacological agents that modulate NCX activity. Concentrations are given below each bar (in µM). Number of knobs tested are indicated in parentheses above each bar. C, Averaged Ca^2+^ responses due to reverse mode activity of NCX from WT (gray) and OMP^−/−^ knobs (black) show a 2.5-fold increase (p<0.0001) in time-to-peak in OMP^−/−^ (*n* = 27; *N* = 5) *vs*. WT mice (*n* = 26; *N* = 4). D, Averaged decay time courses of the same signal in WT (gray) and OMP^−/−^ knobs (black) reveal an approximately 2-fold increase in the recovery rate in OMP^−/−^ mice (p<0.03). Data were normalized to the value obtained immediately at the end of the 20-s low Na^+^ stimulus.

NCX activity was further characterized by monitoring the rise of Ca^2+^_i_ in response to a stepwise reduction of external Na^+^. Reduction of extracellular Na^+^ activates the Ca^2+^ entry mode of NCX with concomitant extrusion of Na^+^ out of the cell accompanied by an elevation of Ca^2+^_i_. When 24 mM external Na^+^ was puffed onto the knobs for 20 s, a robust rise in Ca^2+^_i_ was observed, indicating that NCX was acting in the reverse mode (Figure 4A). To substantiate that the rise in Ca^2+^_i_ was a result of NCX activity, we tested the effects of pharmacological inhibitors. An effective blocker of NCX in its Ca^2+^ entry mode, the isothiourea derivative KB-R7943 (10 µM) [61], effectively and reversibly abolished the Ca^2+^ rise (Figure 4A, B *)*, whereas 5 µM KB-R7943 was ineffective (Figure 4B), confirming the known concentration dependence of this drug. Another potent NCX inhibitor, the amiloride analogue 3,4-dichlorobenzamil (DCB; 10 µM), also diminished the Ca^2+^ influx in a reversible manner (Figure 4A, B). NCX-mediated Ca^2+^ influx is augmented as the cytosolic Na^+^ increases [62]. Therefore, to further characterize the function of NCX we tested the effect of elevating Na^+^_i_ by oubain exposure. Oubain, an inhibitor of the Na^+^/K^+^-ATPase, results in elevated Na^+^_i_. After ouabain (1 mM) treatment, the magnitude of the Ca^2+^ signal was significantly enhanced, by as much as 50–100%, confirming the Na^+^_i_-dependent nature of the Ca^2+^ influx through NCX (Figure 4B). These analyses were repeated in the OMP^−/−^ mice (Figure 4B) and confirmed the functional presence of NCX and its equivalent response to the pharmacological agents in OMP^−/−^ knobs. Thus, the pharmacological analyses illustrate that functional activity of NCX can be probed by manipulating Na^+^_o_, Na^+^_i_, and by specific pharmacological agents.

To test if a deficit in NCX function is responsible for the delayed recovery of elevated Ca^2+^_i_ in the OMP^−/−^ mouse, the time course of Ca^2+^_i_ responses induced by a 20-s pulse of 24 mM external Na^+^, a measure of NCX efficiency, was compared for WT and OMP^−/−^ mice. The Ca^2+^_i_ rise of WT mice reached its peak rapidly, with a slow decay during the pulse (Figure 4C). In contrast, in the OMP^−/−^ knobs, only a slow and steady Ca^2+^_i_ increase was observed that reached its peak almost at the end of the pulse (Figure 4C). The time-to-peak was 2.5-fold slower in the OMP^−/−^ mice (*n* = 26, *N* = 5; *p*<0.0001) compared to WT mice (*n* = 27, *N* = 5), indicating a reduced activity of the Ca^2+^ entry mode of NCX in the OMP^−/−^ knobs. When the low Na^+^ pulse was turned off, the modality of NCX reversed to once again extrude the elevated Ca^2+^_i_. The decay rate measured in the Ca^2+^ efflux mode (Figure 4D) was approximately 2-fold slower in the OMP^−/−^ mouse (20 s Na^+^ stimulation: WT: τ = 3.8 s, *n* = 26, *N* = 5; OMP^−/−^: τ = 8.5 s, *n* = 27, *N* = 5). These results suggest that NCX activity in the OMP^−/−^ mice is reduced in the Ca^2+^ efflux mode as well as in the reverse Ca^2+^ entry mode. To partially decrease the adaptive behavior of the NCX activity in the WT response, 24 mM Na^+^_o_ was also applied for 10 s. The decay rate was again almost 2-fold slower in the OMP^−/−^ mice (*n* = 12, *N* = 4), suggesting that the decay kinetics were not affected by adaptation. Taken together, our data demonstrate that in the absence of OMP a kinetic deficit exists in transporting Ca^2+^ in both directions through NCX, suggesting that OMP serves as a modulator of NCX function in OSN knobs.

### OMP influences NCX activity via CaM and Bex1

**Figure 5:**
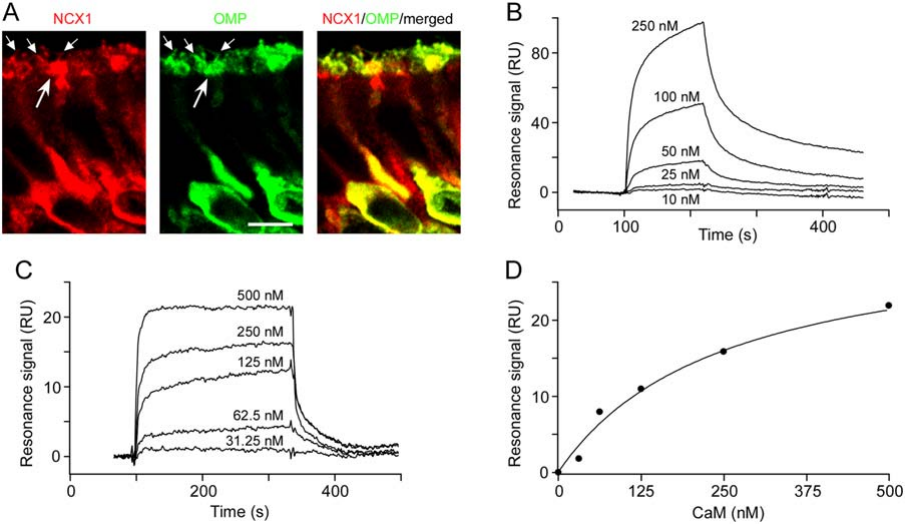
Evidence that OMP regulates NCX1 through interaction with CaM and Bex. A, NCX1 and OMP are highly expressed and co-localized in cell bodies, dendrites, dendritic knobs (long arrows) and cilia (short arrows) of mature OSNs of WT mice. Scale bar, 5 µm. B, Sensorgrams for interaction of XIP peptide immobilized on a CM5 sensorchip and CaM (10, 25, 50,100, 250 nM) using a BIAcore 3000 biosensor. Running buffer used during recording contained 0.1 mM Ca^2+^. Data were fitted with a 1∶1 Langmuir binding model using BIAevaluation software, giving K_d_ = 20 nM. C, Sensorgram for interaction of immobilized Bex1 (50–75) peptide and CaM (31.25, 62.5, 125, 250, 500 nM) on a CM5 chip in presence of 0.1 mM Ca^2+^. D, Because of the rapid on/off kinetics of the interaction between CaM and Bex1 peptide, saturation curves of equilibrium response versus CaM concentration were analyzed using a nonlinear 1∶1 binding isotherm model, giving K_d_ = 280 nM.

NCX protein has been reported on the apical regions of OSNs [59], [63], [64]. *In situ* hybridization indicates that mRNAs for multiple NCX genes are present in OSNs [63], [65], and the NCX1 protein is expressed in nearly all neurons of the olfactory epithelium [63]. To determine whether the influence of OMP on NCX activity is reflected in cellular localization of the two proteins in OSNs we performed double-immunofluorescent staining for NCX1 and OMP on olfactory epithelium of the WT mouse. At high magnification (Figure 5A), it is evident that OMP and NCX1 are co-localized in the OSN cell bodies, dendrites, dendritic knobs and sensory cilia. A similar expression pattern of NCX1 was observed in the OMP^−/−^ mouse (not shown). These observations provide supporting evidence that the compromised ability in reducing the elevated [Ca^2+^]_i_ in OMP^−/−^ mice could rather be attributed to a functional deficit of NCX activity than to a deficit in NCX protein expression.

A possible mechanism by which OMP could influence NCX activity is through a dynamic interaction of OMP and Bex1. Bex1 protein was recently identified as an interacting protein partner of OMP [37]–[40] that binds calmodulin (CaM) in a Ca^2+^-dependent manner [41]. These observations provide the basis for a potential mechanistic link between OMP, Bex protein, CaM and NCX. An interaction between CaM and NCX1 was suggested to be mediated through the exchanger inhibitory peptide (XIP) site on the large intracellular loop of NCX1 [66]. This prompted us to evaluate the interactions of the XIP peptide of NCX1 and the CaM binding region of Bex1 with recombinant calmodulin (CaM) and OMP in real-time using surface plasmon resonance (SPR). To address whether CaM is able to bind to NCX, synthetic XIP peptide (NCX1 amino acids 251–271) was immobilized in flow cells of a CM5 sensor chip, and purified CaM at various concentrations was passed over the surface of the flow cell. CaM bound to the immobilized XIP (Figure 5B) in a Ca^2+^-dependent manner. Using the 1∶1 Langmuir binding model, the K_d_ value for XIP peptide binding to CaM was calculated to be 20±6 nM.

We also confirmed Ca^2+^-dependent interaction of Bex1 peptide with CaM by SPR analysis. CaM interacted with immobilized Bex1 (residues 50–75) rapidly and reversibly (Figure 5C), consistent with our previous report [41]. Due to the fast on/off rate, the affinity value was evaluated using steady-state analysis [67]. The dissociation constant was determined to be 280 nM±83 nM for the Bex1 peptide. The interactions of CaM with each of these peptides were dependent on the presence of Ca^2+^. By contrast, the interaction of both OMP and XIP (K_d_ = 700 nM±98 nM) and OMP and Bex1 peptide (K_d_ = 90±12 µM) were Ca^2+^-independent (not shown).

To further assess the interactions of Bex and XIP with CaM, we evaluated the ability of these peptides to influence the CaM-dependent activation of cAMP-PDE activity [68]. Each of these peptides strongly interferes with the CaM-dependent activation of cAMP-PDE activity in a concentration dependent manner (J. Margolis personal comm.). The XIP peptide is 25–50 fold more effective than is the Bex1 peptide consistent with our SPR data (data not shown). In addition, full-length recombinant Bex1 protein also effectively inhibits the CaM-dependent activation of the cAMP-PDE activity (J. Margolis personal comm.). These observations are consistent with a model in which OMP influences NCX activity in OSN knobs by a dynamic series of multi-protein interactions between OMP, Bex1 and CaM that result in alterations of the efficacy of CaM. Our observations thus also extend prior reports that NCX1 activity is regulated as a multi-protein macromolecular complex [69], [70].

## Discussion

Several major results emerge from this study. (1) Ca^2+^ imaging of the dendritic knobs of OSNs from OMP^−/−^ mice shows that the lack of OMP compromises rapid extrusion of elevated Ca^2+^_i_ generated in response to activation of the prototypic cAMP signalling pathway that is used in canonical olfactory sensory neurons for signal transduction. (2) The findings indicate that this effect depends on altered regulation of NCX activity in the OSN dendritic knobs of OMP^−/−^ mice. (3) Molecular analysis indicates that proper Ca^2+^ extrusion might depend on a series of protein-protein interactions among OMP, Bex1, Ca^2+^/CaM and NCX, and that these interactions could be altered in OSNs from OMP^−/−^ mice. (4) Together, these findings provide new insight into the mechanisms underlying Ca^2+^ regulation in dendritic knobs of mouse OSNs.

OMP is known to act as a modulator of olfactory signal transduction as demonstrated by the electrophysiological and behavioral deficits of OMP^−/−^ mice [12]–[15] and by the rescue of these deficits with an OMP-expressing adenovirus [10], [11]. In the present report we have identified a potential site in the signal transduction cascade where OMP acts and provide evidence for a mechanism by which this occurs. Our demonstration that the recovery of Ca^2+^ transients was significantly slower in the OMP^−/−^ mouse illustrates that OMP plays an important role in the processes of Ca^2+^ removal in the dendritic knob as distinct from the various mechanisms of Ca^2+^ entry through CNG channels, voltage-gated Ca^2+^ channels, or release from Ca^2+^ stores (Figure 1). Furthermore, our data demonstrate that the mechanism of Ca^2+^ removal is by way of extrusion primarily via NCX (Figure 2), which is abundantly present in the dendritic knobs (Figure 4), and not by intracellular Ca^2+^ re-uptake. We found no significant difference in resting level of Ca^2+^_i_ between WT and OMP^−/−^ mice that might directly influence other steps in the transduction cascade.

To demonstrate functional deficiency of NCX activity in the absence of OMP, we took advantage of the bi-directional nature of NCX that can transport Ca^2+^ into, or out of, cells depending on the relative concentrations of Ca^2+^ and Na^+^ (Figure 3). The highly significant kinetic deficit in transporting Ca^2+^ through NCX in OSN dendritic knobs of OMP^−/−^ mice indicates that OMP facilitates NCX activity. We propose a mechanism by which this occurs: OMP influences NCX activity in OSNs by a series of multi-protein interactions involving OMP, Bex1 and CaM. We have previously reported that OMP interacts with Bex1 [37], [38]; that this interaction preferentially takes place with a short-lived OMP-dimer [39]; and that the Bex1 protein interacts with CaM in a Ca^2+^-dependent manner [41]. The eXchanger Inhibitory Peptide (XIP) region (residues 251–271) of NCX1 specifically inhibits NCX exchange activity, interacts with CaM [66] and is a high affinity CaM binding site (K_d_ = 20 nM) as confirmed by our SPR experiments (Figure 5). Furthermore, OMP can interact with the XIP peptide as well as with Bex1 implicating OMP as a modulator of CaM dependent processes in the transduction cascade.

Thus the interaction of the XIP site of NCX with CaM and with OMP provides a linkage between OMP and NCX through the interactions of OMP, XIP, Bex1 and CaM. Consistent with this postulate is our recent observation that odor-evoked field potential responses (EOG) of the Bex1^−/−^ mouse show a delayed onset and recovery time course (Leinders-Zufall and Margolis, unpub.) similar to that reported for OMP^−/−^ mice [10], [15]. The involvement of CaM in almost every step of the olfactory signal transduction pathway [35], [71] suggests that other steps of this pathway should also be influenced in the OMP-KO. This is apparent from the delayed onset and prolonged recovery in response to stimulus application observed in the EOG recordings of the OMP-KO [10], [15] as well as the altered responses seen in voltage-gated dye recordings in response to stimulus application to the olfactory epithelium of OMP-KO mice [12]. Curiously, our data indicating that OMP influences a very late step in the transduction cascade might appear to be in disagreement with the conclusion by Reisert et al. [9] that OMP acts at a very early step in the cascade to regulate cAMP kinetics. In that study single cell recordings of dissociated OSNs of OMP-KO mice were monitored using suction electrodes, a method thought to report primarily from events occurring in the cilia. However, rather than being discordant with our interpretations, their data are consistent with our hypothesis that the interaction of OMP and Bex can modulate multiple CaM-mediated events throughout the transduction cascade, and with the previous reports [10], [15] that both early and late events in the EOG are altered in the OMP-KO mouse. These observations are consistent with the complex role of calmodulin that influences multiple steps in the olfactory transduction pathway. It is also consistent with the recent reports [52], [53] of a significant role for PMCA, another CaM regulated participant in the transduction process.

In conclusion, we have demonstrated that OMP plays an important role in olfactory signal transduction by influencing the rate of removal of elevated Ca^2+^_i_ in OSN dendritic knobs. The persistently delayed restitution of Ca^2+^ transients in the OSN knobs of OMP^−/−^ mice, demonstrates that OMP participates in the regulation of NCX activity. In summary, we propose a model, and provide evidence, to explain the involvement of OMP in the regulation of NCX activity that involves the interaction of OMP, Bex and CaM to regulate NCX activity. Further, we suggest that the dynamic interactions among these proteins provides a mechanism to help explain the complex phenotype of the OMP-KO mouse based on OMP modulation of the efficacy of CaM at multiple steps of the olfactory chemo-transduction pathway. This provides the first mechanistic insight to the elusive role of OMP and provides a framework for further investigation of its function.

## Materials and Methods

### Confocal Ca^2+^ imaging using intact mouse MOE preparation

Mice (3–6 month old) of homozygous OMP^−/−^ mice or the parental strain (129X1/Sv; Jackson Laboratories, Bar Harbor, ME) were used. The generation and genotyping of the OMP-null mice has been described previously [15]. All procedures were approved by the Institutional Animal Care and Use Committee of the University of Maryland School of Medicine. The main olfactory epithelium (MOE) was removed from the nasal septum and mounted in a recording chamber with the mucous layer facing up [72], [73]. The preparation was superfused continuously at room temperature with normal oxygenated external solution (95% O_2_/5% CO_2_) containing (in mM) 120 NaCl, 25 NaHCO_3_, 5 KCl, 5 BES, 1 MgSO_4_, 1 CaCl_2_, and 10 mM glucose. The epithelial preparation was loaded with the Ca^2+^ indicator fluo-4/AM [74], [75]. Changes in intracellular Ca^2+^ concentration were imaged in single knobs by using a confocal laser system [74], 75. Optical sections were ∼9 µm thick. Images were acquired at rates between 0.63 and 0.45 Hz using BioRad's LaserSharp software and analyzed using NIH Image 1.63 and Igor Pro software (Wavemetrics, Lake Oswego, OR). Data are expressed as mean±SEM with the number of knobs (*n*) and mice (*N*) indicated. Unless otherwise stated, Ca^2+^ transients shown are averaged traces from 5 representative knobs. Statistical significance between two groups was evaluated using the Student's unpaired t-test. Probability values (*p*) of <0.05 were considered statistically significant.

Stimuli were diluted in extracellular solution [in mM: 145 NaCl, 5 KCl, 1 CaCl2, 1 MgCl2, 10 Hepes, pH = 7.3 (NaOH), 300 mOsm (glucose)] immediately before use and focally ejected onto the olfactory knobs by air pressure and using multi-barrelled stimulation pipettes. Low external Ca^2+^ solution contained (in mM): 120 NaCl, 25 NaHCO3, 5 KCl, 4.25 CaCl2, 5 EGTA, 1 MgCl2, 5 BES, and 6 glucose at pH = 7.3 and 300 mOsm, giving a free Ca^2+^ concentration of approximately 0.6 µM. Low external Na^+^ solution contained (mM): 125 LiCl, 20 NaCl, 5 KCl, 1 CaCl2, 1 Mg Cl2, 10 Hepes, 4 NaOH, pH = 7.3, 300 mOsm (glucose). 3-isobutyl-1-methylxanthine (IBMX), KB-R7943 (kindly provided by Nippon Organon, Inc., Osaka, Japan), 3,4-dichlorobenzamil hydrochloride, and 5,6-carboxyeosin diacetate, succinimidyl ester (CE; Invitrogen, Eugene, OR) were prepared in DMSO. Dilutions were made freshly with final DMSO concentrations of <0.1% (v/v). Unless otherwise stated, chemicals were obtained from Sigma (St. Louis, MO).

Carboxyeosin (CE) stock solution, initially made in DMSO, was added to the extracellular solution (for composition see above) to give a concentration of 10 µM. The tissue was preincubated for 15 minutes and then superfused with CE-free solution for an additional 10 minutes to allow de-esterification of CE and to wash off the residual compound in the bath. The concentration of 10 µM CE is 500-fold higher than the reported IC50 value [76].

KB-R7943 and DCB (3,4-dichlorobenzamil hydrochloride) were both first dissolved in DMSO and then further diluted in extracellular solution immediately before use to yield a concentration of 5–10 µM and 10 µM, respectively. Both KB-R7943 and DCB were directly applied to the knobs 5 minutes prior and during additional measurements. DCB was also tested at a concentration of 20 µM. The effect of 10 or 20 µM DCB was indistinguishable, except at the higher concentration where the inhibitory effect of DCB was irreversible. The general inhibitory working concentration of DCB on NCX used by several authors is 10–40 µM in various systems [e.g. [77]–[79]].

Thapsigargin (Tg) was initially dissolved in DMSO to give a 2 mM stock solution. The agent was diluted to the final concentration of 200 nM immediately before use, sonicated, and applied to the extracellular solution for 15 minutes. In some experiments recordings were made from the same knob before and after loading with Tg.

For a few selected experiments, we used freshly dissociated mouse OSNs by adapting previously described procedures [80]. In this case the septal MOE was minced into small pieces, incubated for 15 min at 37°C in low Ca^2+^ solution containing papain (1.4 mg/ml) and DNase (1 U/ml; Promega). The tissue was then gently extruded in normal oxygenated external solution and centrifuged for 10 min at 600 rpm. The pellet was resuspended in external solution and the released single cells were plated onto a glass cover slip previously coated with 0.01% poly-L-lysine and 0.01% laminin. Resting Ca^2+^concentrations were determined by imaging olfactory knobs using the *in situ* preparation or freshly dissociated OSNs. Single-wavelength calibration (fluo-4/AM) was performed as described by Kao et al. [81] and Leinders-Zufall et al. [29]. Dual-wavelength calibration (fura-2/AM) was performed in dissociated OSNs by using a microscope system equipped with UV laser and CCD camera (kindly provided by Dr. J.P.Y. Kao, University of Maryland, Baltimore). The following equation was used to determine resting Ca^2+^_i_ in fura-2/AM loaded OSNs:
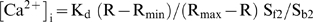
where K_d_ is the Ca^2+^-fura-2 dissociation constant, 224 nM. R is (w_1_−w)/(w_2_−w) where w_1_ and w_2_ are the fluorescence intensity at excitation wavelength of 340 and 380 nm, respectively, and w equals autofluorescence after cell lysis with digitonin (50 µM). Some of the values were predetermined by Dr. Kao for this specific imaging system: R_min_ = 0.358±0.0585; R_max_ = to4.69±0.678; S_f2_/S_b2_ = 4.072±0.533.

### Colocalization of OMP and NCX1 by immunofluorescent staining

Olfactory neuroepithelial tissue was obtained from postnatal day 7 (P7) WT mice. Mice were anesthetized with 60 mg/kg nembutal and perfused transcardially with 20 ml of ice cold PBS followed by 30 ml of 4% freshly prepared phosphate buffered paraformaldehyde. Tissue was dissected and postfixed for two hours in cold fixative and cryoprotected overnight in 30% sucrose at 4°C. Tissues were embedded in OCT (Tissue Tek, Sakura, Torrance, CA) and snap-frozen in a dry ice/isopentane bath. Coronal cryostat sections of olfactory tissue (12 µm) were attached to Superfrost-plus microscope slides (Fisher, Pittsburgh, PA), dried at 37°C for 15 min and stored at −80°C until needed. For single and double-labeled immunofluorescence staining goat anti-OMP antibody (1∶20,000, [3] and rabbit anti-NCX1 antibody (1∶2,000, Swant, Bellinzona, Switzerland) were applied to the olfactory turbinate sections on slides and processed essentially as described previously [40].

Anti-OMP staining used a polyclonal goat primary antiserum generated against OMP purified from rat olfactory tissue [3]. This antiserum has been characterized extensively for immunocytochemistry [e.g. [2], [7], and its specificity has been verified by the absence of immunoreactivity in OMP^−/−^ mice [1].

Anti-NCX1 staining used a rabbit polyclonal anti-NCX1 antiserum directed against canine cardiac sarcolemmal NCX1 originally reported by Philipson et al. [82]. This antiserum gives the same staining pattern in mouse olfactory epithelium [83] as does the anti-NCX1-peptide antiserum AbO-8 [84]. Additional characterization of this antiserum has been published elsewhere [85], [86].

### Surface plasmon resonance (SPR) experiments

The Biacore 3000 (Biacore AB, Uppsala, Sweden) was used to study peptide-protein interactions by SPR. Bovine calmodulin was purchased from Sigma, recombinant rat OMP was expressed and purified as previously described [87]. The XIP peptide (NCX1 251–271, RRLLFYKYVYKRYRAGKQRGM) and the CaM binding peptide of Bex1 (Bex1 50–75, RGGRRRFRVRQPIAHYRWDLMQRVGE) were synthesized and HPLC purified in the biopolymer/genomics core facility of the University of Maryland. Each peptide was separately immobilized on a carboxymethylated-dextran (CM5) chip surface using standard carbodiimide chemistry according to the manufacturer's instructions. The average amount of each immobilized peptide was about 700 RU (resonance unit). Binding was evaluated over a concentration range of 0–500 nM CaM or OMP in running buffer in the absence (NaCl 150 mM; HEPES 10 mM; pH 7.4) or presence of Ca^2+^ (NaCl 150 mM; HEPES 10 mM; CaCl_2_ 0.1 mM; pH 7.4) under a continuous flow of 5 µl/min for 2 minutes, unless otherwise stated. Flow cells were regenerated by flushing with running buffer without CaCl_2_. Binding data were fitted and analyzed using Biacor evaluation version 4.1 software (Biacore). Data are representative of three separate experiments.
